# Polypharmacy among children and adolescents with psychiatric disorders in a mental referral hospital in Botswana

**DOI:** 10.1186/s12888-017-1347-6

**Published:** 2017-05-10

**Authors:** Anthony A. Olashore, Ambrose Rukewe

**Affiliations:** 1 Department of Psychiatry, University of Botswana, Gaborone, Botswana; 2 Department of Anesthesia and Critical Care, University of Botswana, Gaborone, Botswana

**Keywords:** Polypharmacy, Children and adolescents, Psychiatric disorders

## Abstract

**Background:**

There is a dearth of data on polypharmacy in child and adolescent mental health in Africa, especially Botswana where children and adults are treated in the same facility by general adult psychiatrists. This study was therefore designed to assess the prevalence and the risk factors of psychiatric polypharmacy among children and adolescents treated at Sbrana Psychiatric Hospital, Lobatse, Botswana.

**Methods:**

Data involving socio-demographics, diagnosis (using ICD-10 classification) and pharmacological treatment were retrieved from the records of 120 children and adolescents aged below 18 years, between 1 January 2012 and 31 July 2016, who presented with psychiatric disorders. They were analysed with univariate and multivariate models.

**Results:**

The prevalence of psychiatric polypharmacy was 29.2%. Psychiatric co-morbidity (OR = 3.374, 95% CI: 1.177–9.9673) and psychotropic side effects (OR = 5.782, 95% CI: 1.636–20.430) were significantly associated with polypharmacy after regression analysis.

**Conclusion:**

Psychiatric co-morbidity and psychotropic side effects were significant risk factors for polypharmacy in Botswana.

## Background

Psychopharmacological treatment in the child and adolescents with psychiatric disorder is increasingly becoming a matter of concern both in the western worlds and developing countries [1, 2]. Of greater concern is the rate of off-label use of psychotropic medication and polypharmacy especially in the developing countries [2] where the practice of specialized care is still very rudimentary. Some patients might present with more than one psychiatric diagnosis or resistance to monotherapy, necessitating the use of multiple psychotropic medications or other non-evidenced based methods of care [3–5]. Other instances are the use of another medication to treat the adverse reaction of an effective agent or adjunctive use of medications (adjunctive polypharmacy) and augmentation (augmentation polypharmacy) [3, 4, 6, 7]. In psychiatry, polypharmacy has been defined by the National Association of State Mental Health Program Directors (NASMHPD) as the use of two or more psychiatric medications in the same patient [6, 7]. NASMHPD described other types of polypharmacy: *“Same-class polypharmacy”*, where agents from the same class are used for the same symptoms, e.g., use of two types of Phenothiazines in a case of psychosis; *“Multi-class polypharmacy”*, use of more than one agents from different classes for the same disorder, e.g., carbamazepine and olanzapine for mania; and *“Total polypharmacy”* which is described as ‘total drug load’ including non-psychotropic medications e.g., ibuprofen for co-morbid aches [4, 6, 7].

There is a high prevalence of psychiatric polypharmacy in child and adolescents ranging from 2.9–27% worldwide [8, 9]. Another study from India, with a smaller sample size among inpatients reported a rate of 52% [10]. While there is little evidence to support this practice, there are growing concerns on the adverse effect of combining two or more medications [3, 4]. It compounds regimens complexity leading to non-compliance to treatment and increases the effects of drug interaction. Some medications affect liver enzymes, hence the bio-availability and therapeutic effect of drugs. For example, carbamazepine which is an enzyme inducer may reduce the effectiveness of some antipsychotics [3, 4]. Moreover, the fact that children often need longer duration of treatment exposes them to more adverse effects of polypharmacy or drug interaction [2, 3, 9].

To the best of our knowledge, there is a dearth of data on polypharmacy in child and adolescent mental health in Africa, especially Botswana where children and adults are treated in the same facility by general adult psychiatrists. This study was designed to assess the prevalence and the risk factors of psychiatric polypharmacy among children and adolescents treated at our referral hospital between 1 January 2012 and 31 July December 2016. This we believe will add more weight to the on-going advocacy on the need for specialized care for children and adolescents in Botswana.

## Methods

After obtaining institutional ethics approval, we reviewed the records of children and adolescents aged below 18 years between 1 January 2012 and 31 July 2016 who presented with psychiatric disorders. The study was conducted in Sbrana Psychiatric Hospital (SPH) Lobatse, located in the south western district of Botswana. It is the only tertiary mental institution in the country with facilities to cater for all general adult as well as paediatric psychiatric patients, and it accepts all types of mental disorders, ranging from minor to severe conditions. Data were retrieved from the patients’ medical records comprising socio-demographics, diagnosis and treatments (including follow-up notes) using a form designed by the authors. The diagnosis was extracted from the most prominent and consistent group of symptoms using ICD-10 classification and as indicated by the members of the multidisciplinary care teams. Only subjects who had pharmacotherapy alone or both pharmacotherapy and psychotherapy were studied. Polypharmacy was only considered when two or more psychotropic medications (i.e., antipsychotics, antidepressants, anticholinergics, stimulants, mood stabilizers, etc.) were used at the same treatment period (i.e., same class, adjunctive, multi-class and augmentation polypharmacy), not total polypharmacy. We excluded cases where switching of psychiatric medications were established from documentation, either in the same episode or different episodes. We also excluded patients who received only psychotherapy such as counselling, psycho-education and cognitive behavioral therapy. Out of a total of 238 patients treated during the study period; 118 who had only psychotherapy were excluded. The disorders were grouped into externalizing conditions (conduct disorder and hyperkinetic disorder), internalizing (anxiety disorder and mood disorders) and others (epilepsy, enuresis, somatoform disorder, psychosis, conversion disorder, etc). We used age 10 years as cut-off between the younger child and older child for our analysis.

Data Analysis was done using the Statistical Package for Social Sciences (SPSS for windows 16.0, SPSS Inc., Chicago, IL, USA). Frequency tables were employed for descriptive statistics such as socio-demographics and clinical variables. Cross-tabulations were done to show the relationships between relevant socio-demographics (such as age group, birth order), clinical variable and polypharmacy. Chi-square test was used to determine associations between the categorical variables. Predictor variables (i.e., independent factors) for identified dependent variables which is polypharmacy was further investigated using binary logistic regression analysis. The level of statistical significance for all tests was set at *p* < 0.05.

## Results

Only 120 subjects met the inclusion criteria. The mean age (sd) of the subjects was 11.9 (4.4) years (67.5% males, 32.5% females). Forty-six (38.3%) were 10 years and below (Table 1).

**Table 1:** Socio-demographic characteristics of children and adolescents with psychiatric disorders in Sbrana Psychiatric Hospital Lobatse, Botswana, January 2012 to July 2016

Variable	N (%)
Age (years, M = 11.94, SD = 4.4)	
Age group	120 (100)
≤ 10	46 (38.3)
>10 years	74 (61.7)
Gender	120 (100)
Male	81 (67.5)
Female	39 (32.5)
Birth order^a^	115 (100)
First child	41 (35.7)
Others	74 (64.3)

^a^N not equal to 120 due to missing data

The disorder grouped as others comprising epilepsy, enuresis, somatoform disorder, psychosis, conversion disorder, etc. had the highest proportion 70 (58.3%), followed by externalizing disorders 43 (35.8%) and internalizing disorders 7 (5.8%). Sixty-nine (57%) patients had co-morbid psychiatric disorders while 35 (29.2%) had 2 or more psychotropic medications (polypharmacy) (Table 2).

**Table 2:** Detailed clinical variables in children and adolescents with psychiatric disorders in Sbrana Psychiatric Hospital Lobatse, Botswana, January 2012 to July 2016

Variables	N (%)
Disorders	120 (100)
Externalizing disorders	43 (35.8)
Internalizing disorders	7 (5.8)
Other disorders	70 (58.3)
Past psychiatric history	120 (100)
Absent	24 (20)
Present	96 (80)
Past medical history	120 (100)
Absent	109 (90.8)
Present	11 (9.2)
Psychiatric co-morbidity	120 (100)
Absent	51 (42.5)
Present	69 (57.5)
Physical/medical comorbidity	120 (100)
Absent	106 (83.3)
Present	14 (11.7)
Mode of care	120 (100)
In-patient	31 (25.8)
Out-patient	89 (74.2)
Prescribing pattern	120 (100)
Monotherapy	85 (70.8)
Poly-therapy	35 (29.2)

The factors with significant association with polypharmacy were age group (χ^2^ = 5.319, *p* = 0.021), psychiatrist care (*χ* ^*2*^ = 5.772, *p* = 0.016), psychiatric co-morbidity (χ^2^ = 7.802, *p* = 0.005) and psychotropic side effect (*χ* ^*2*^ = 6.640, *p* = 0.010) using Chi-square test (Table 3). The logistic regression analyses indicated that the significant risk factors of polypharmacy were psychiatric co-morbidity (odds ratio [OR] 3.374, 95% confidence interval [CI] 1.177–9.673, *p* = 0.024) and psychotropic side effects (OR 5.782, 95% CI: 1.636–20.430, *p* = 0.006) (Table 4), after adjusting for all factors that were significant as well as birth order and diagnosis, which slightly fell short of being significant, with *p* values of 0.063 and 0.068 respectively, on Chi-square test.

**Table 3:** Association between clinical variables and polypharmacy in children and adolescents with psychiatric disorders in Botswana, January 2012 to July 2016

Risk factors	Mono –pharmacy N (%)	Polypharmacy N (%)	*df*	χ^2^	*p*
Age group					
≤10	27 (58.7)	19 (41.3)	1	5.319	*0.021*
>10	58 (78.4)	16 (21.6)			
Gender					
Female	29 (74.4)	10 (25.6)	1	0.348	0.555
Male	56 (69.1)	25 (30.9)			
Birth order					
First born	16(39.0)	25(61.0)	1	3.322	0.068
Others	17(23.0)	57(77.0)			
Past psychiatric history					
Absent	14 (58.3)	10 (41.7)	1	2.269	0.132
Present	71 (74.0)	25 (26.0)			
Medical history					
Absent	78 (71.6)	31 (28.4)	1	0.307	0.582
Present	7 (63.6)	4 (36.4)			
Psychiatrist care					
Not given	64 (66.0)	33 (34.0)	1	5.772	*0.016*
Given	21 (91.3)	2 (8.7)			
Psychiatric co-morbidity					
Absent	43 (84.3)	8 (15.7)	1	7.802	*0.005*
Present	42 (60.9)	27 (39.1)			
Physical co-morbidity					
Absent	77 (72.6)	29 (27.4)	1	1.438	0.230
Present	8 (57.1)	6 (42.9)			
Diagnosis					
Externalizing disorders	26 (60.5)	17 (39.5)	2	5.524	0.063
Internalizing disorders	7 (100)	-			
Others	52 (74.3)	18 (25.7)			
Psychotropic side effect					
Absent	75 (75.8)	24 (24.2)	1	6.640	*0.010*
Present	10 (47.6)	11 (52.4)			
Mode of care					
In-patient	22 (71.0)	9 (29.0)	1	0.000	0.985
Out-patient	63 (70.8)	26 (29.2)			
Type of intervention					
Only Pharmacological	30 (68.2)	14 (31.8)	1	0.28	0.866
Both Psychological and pharmacological	46 (69.7)	20 (30.3)			

Significant *p*-value in italics, χ^2^ = Chi-square, *df* degree of freedom, *p* = *P* value

**Table 4:** Factors associated with polypharmacy

Risk factors	Wald	*df*	*p*-value	OR	95% CI	
					Lower	Upper
Age group^a^	.850	1	.356	1.786	.521	6.124
Diagnosis^b^	.001	1	.974	1.021	.291	3.587
Psychiatrist care^c^	1.557	1	.212	.347	.066	1.829
Birth order^d^	3.503	1	.061	.376	.135	1.047
Psychiatric comorbidity^e^	5.122	1	*.024*	3.374	1.177	9.673
Psychotropic side effect^f^	7.424	1	*.006*	5.782	1.636	20.430

Significant test of association in *italics, OR*  odds ratio, *CI*  confidence interval, *df*  degree of freedom

^a^> 10 years

^b^Internalizing disorders

^c^Psychiatrist care given

^d^Others

^e^Absent

^f^Absent

## Discussion

The prevalence of polypharmacy (29.2%) in this study is slightly outside the range of 2.9–27% reported in a systematic review by Toteja et al. [11]. It is about two-fold the rate reported in a US study (13.8%) among paediatric patients at discharge from hospitalization, this is not surprising as our series covered in-patient and outpatient services [11]. It is much less than the 52.4% reported in an Indian study and 51% by Dean et al. in Australia about a decade ago [10, 12]. However, our rate of 41.3% in the younger child compared to 21% in the older was in direct opposition to the trend; lower rates were reported in the younger child than adolescents [11]. We cannot explain this difference given that the general adult psychiatrists employed at our centre treat mainly adults with mental health conditions and lack the expertise for complete treatment plan in children and adolescents, which prioritizes developmental and psychosocial interventions over psychotropic medications [13, 14]. Our results do indicate that polypharmacy is a common practice in our centre as in other parts of the world [1, 2, 15].

The mean age of our sample 11.9 (4.4) years and a preponderance of male children (67.5%) was comparable to the mean of Toteja et al.’s systematic review of 15 studies that involved 58,041 subjects [11]. We differed in the most commonly reported diagnoses: ours were those grouped under “others” such as epilepsy, enuresis, somatoform disorder, psychosis, conversion disorders, etc., while they reported more externalizing mental conditions hyperkinetic disorder and conduct disorder. Whereas mood disorders (internalizing conditions) accounted for the mostly treated mental illnesses with polypharmacy in the Indian study earlier mentioned [10]. Our finding of 5.8% with internalizing disorders is too small a sample to derive meaningful conclusions. The pattern of presentation of psychiatric cases to our treatment centre restricts us to these groupings for convenience. These differences however highlight the complexity of child and adolescent mental disorders involved in polypharmacy all over the world.

An initial analysis did not find significant association with gender and diagnosis in keeping with previous studies [10, 11]. Our data showed that age group, psychiatrist care, psychiatric co-morbidity and psychotropic side effects influenced the rate of polypharmacy in our series. These variables including diagnosis were further analyzed with the logistic regression. Our results indicated that psychiatric co-morbidity and psychotropic side effects were significantly associated with the presence of polypharmacy as their odds ratio were high contrary to the findings of Russell et al. [10]. However, other studies agreed with our findings that co-morbidity and more complex presentation (diagnosis that are not clear-cut) significantly correlate with the risk of polypharmacy [12, 15, 16]. The pressure to quickly resolve patients’ symptoms which make non-pharmacological options less attractive and favour an increase in polypharmacy with attendant psychotropic side effects have been reported by other investigators [17, 18].

Being a retrospective study, we could not explore if other risk factors were involved in polypharmacy in our centre. Another limitation of this study is the small number of subjects, which imposes restriction on the external validity of our results. We admit that while cases where switching of psychiatric medications were clear-cut from documentation were excluded; given the retrospective design, few cases could have been missed. There were some missing data: birth order as a variable was omitted for some patients which could have impacted our result. A prospective, long-term study would address these concerns. Despite these constraints, we believe this study has highlighted the practice of polypharmacy in paediatric psychiatric patients in Botswana and the need for specialized care of this population.

## Conclusions

In conclusion, the prevalence of psychiatric polypharmacy was 29.2% among children and adolescents with psychiatric disorders in Botswana; co-morbidity and psychotropic side effects were significantly associated with the presence of polypharmacy.

## Abbreviations

ICD 10: International Classification of Diseases version 10
SPH: Sbrana Psychiatric Hospital

## References

[1] Rao P, Zepf FD, Chakrabarti I, Sigalas P. Atypical antipsychotic prescribing patterns amongst Child and Adolescent Mental Health Services clinicians in a defined National Health Service Trust, Translational Developmental Psychiatry. 2016;4:1, 28537. Available at http://dx.doi.org/10.3402/tdp.v4.28537.

[2] Vitiello B. Principles in using psychotropic medication in children and adolescents. IACAPAP e-textbook of child and adolescent mental health. Geneva, Switzerland: International Association for Child and Adolescent Psychiatry and Allied Professions. Retrieved from http://iacapap.org/wp-content/uploads/A. 2012.

[3] Stahl SM (2002). Antipsychotic polypharmacy: evidence based or eminence based?. Acta Psychiatr Scand.

[4] Kukreja S, Kalra G, Shah N, Shrivastava A (2013). Polypharmacy in psychiatry: a review. Mens sana monogr.

[5] Walkup J (2009). Practice parameter on the use of psychotropic medication in children and adolescents. J Am Acad Child Adolesc Psychiatry.

[6] Flaum M, Karp S, Parks J. Technical report of psychiatric polypharmacy. A series of technical reports prepared by the: National Association of State Mental Health Program Directors (NASMHPD). 2001:1–24. Available at https://www.nasmhpd.org/sites/default/files/Polypharmacy.pdf.

[7] Adeponle AB, Obembe AO, Adeyemi SO, Suleiman GT (2007). Polypharmacy in psychiatric outpatient practice in northern Nigeria. Afr J Psychiatr.

[8] Saldaña SN, Keeshin BR, Wehry AM, Blom TJ, Sorter MT, DelBello MP, Strawn JR (2014). Antipsychotic polypharmacy in children and adolescents at discharge from psychiatric hospitalization. Pharmacotherapy.

[9] Comer JS, Olfson M, Mojtabai R (2010). National trends in child and adolescent psychotropic polypharmacy in office-based practice, 1996-2007. J Am Acad Child Adolesc Psychiatry.

[10] Russell PS, George C, Mammen P (2006). Predictive factors for polypharmacy among child and adolescent psychiatry inpatients. Clin Pract Epidemiol Ment Health.

[11] Toteja N, Gallego JA, Saito E, Gerhard T, Winterstein A, Olfson M, Correll CU (2014). Prevalence and correlates of antipsychotic polypharmacy in children and adolescents receiving antipsychotic treatment. Int J Neuropsychopharmacol.

[12] Dean AJ, McDermott BM, Marshall RT (2006). Psychotropic medication utilization in a child and adolescent mental health service. J Child Adolesc Psychopharmacol.

[13] Winters NC, Pumariega A, on Community-Based TW, of Care S (2007). Practice parameter on child and adolescent mental health care in community systems of care. J Am Acad Child Adolesc Psychiatry.

[14] American Academy of Child and Adolescent Psychiatry. Policy Statement: Prescribing Psychoactive Medication for Children and Adolescents. Available at https://www.aacap.org/App_Themes/AACAP/docs/clinical_practice_center/systems_of_care/AACAP_Psychotropic_Medication_Recommendations_2015_FINAL.pdf. Revised and Approved on September 20, 2001. Accessed 27 Sept 2011.

[15] McIntyre RS, Jerrell JM (2009). Polypharmacy in children and adolescents treated for major depressive disorder: a claims database study. J Clin Psychiatry.

[16] Fontanella CA, Bridge JA, Campo JV (2009). Psychotropic medication changes, polypharmacy, and the risk of early readmission in suicidal adolescent inpatients. Ann Pharmacother.

[17] Vitiello B (2008). An international perspective on pediatric psychopharmacology. Int Rev Psychiatry.

[18] Díaz-Caneja CM, Espliego A, Parellada M, Arango C, Moreno C (2014). Polypharmacy with antidepressants in children and adolescents. Int J Neuropsychopharmacol.

